# Attention Control and Audiomotor Processes Underlying Anticipation of Musical Themes while Listening to Familiar Sonata-Form Pieces

**DOI:** 10.3390/brainsci12020261

**Published:** 2022-02-13

**Authors:** Chia-Wei Li, Chen-Gia Tsai

**Affiliations:** 1 Department of Radiology, Wan Fang Hospital, Taipei Medical University, Taipei 116, Taiwan; ffiln0628@gmail.com; 2 Graduate Institute of Musicology, National Taiwan University, Taipei 106, Taiwan; 3 Neurobiology and Cognitive Science Center, National Taiwan University, Taipei 106, Taiwan

**Keywords:** prediction, attention, auditory imagery, motivation, motor simulation, musical theme

## Abstract

When listening to music, people are excited by the musical cues immediately before rewarding passages. More generally, listeners attend to the antecedent cues of a salient musical event irrespective of its emotional valence. The present study used functional magnetic resonance imaging to investigate the behavioral and cognitive mechanisms underlying the cued anticipation of the main theme’s recurrence in sonata form. Half of the main themes in the musical stimuli were of a joyful character, half a tragic character. Activity in the premotor cortex suggests that around the main theme’s recurrence, the participants tended to covertly hum along with music. The anterior thalamus, pre-supplementary motor area (preSMA), posterior cerebellum, inferior frontal junction (IFJ), and auditory cortex showed increased activity for the antecedent cues of the themes, relative to the middle-last part of the themes. Increased activity in the anterior thalamus may reflect its role in guiding attention towards stimuli that reliably predict important outcomes. The preSMA and posterior cerebellum may support sequence processing, fine-grained auditory imagery, and fine adjustments to humming according to auditory inputs. The IFJ might orchestrate the attention allocation to motor simulation and goal-driven attention. These findings highlight the attention control and audiomotor components of musical anticipation.

## 1. Introduction

Arousal and attention to reward cues play a substantial role in human reward pursuit. Spectators on the golf course focus on the long putt and almost forget to breathe. When listening to music, people tend to be excited by the musical cues prior to rewarding passages. For example, the peak pleasurable experiences of electronic dance music (EDM) are linked to two musical sections: the build-up and the ensuing drop. The rewarding drop section was found to elicit maximal skin conductance responses in listeners, while some listeners experienced chills and an urge to move their bodies during the final bars of the build-up section that announce the coming of the drop [1]. The antecedent cues of musical rewards are associated with psychological ‘wanting’ or the anticipatory phase of reward processing, leading to increasing activity in reward-related brain regions in the listeners [2,3].

The anticipation of a salient musical passage may entail audiomotor processes irrespective of its emotional valence. Tracking the sequence of antecedent cues and the salient passage may be performed via motor simulation such as covert humming along with the music, especially when the listener is familiar with the piece. This is analogous to the anticipation of the motion of a table tennis ball, in which anticipation performance is based on the recruitment of sensorimotor representations in the premotor cortex [4]. Although a humming voice never amounts to real music, the associated audiomotor representation provides a prediction of the ensuing passage [5]. A salient musical passage may be the main tune of a musical piece. Given that the encoding, storage, and retrieval of tunes are all essential for a number of human social activities [6], it is likely that successful prediction based on cue-triggered auditory imagery of salient tunes is rewarded with pleasure in the brain.

One kind of salient event in music is the recurrence of a main theme, which is often easy to memorize and imagine. In Western classical music, the main theme’s recurrences manifest significant structural and emotional effects. Notably, the musical cues immediately before the main theme’s recurrences are designed to foster the listener’s motivation and cognitive organization towards anticipating the main theme. Using functional magnetic resonance imaging (fMRI), the present study aimed to identify the neural correlates of the cued anticipation of the main themes during listening to familiar music. All musical stimuli in the present study were selected from compositions written in sonata form. Developed in the late eighteenth century, the sonata form became one of the most important forms in Western classical music. The most dramatic moment of this form is typically around the coincidence of the main theme’s recurrence and the harmonic resolution at the start of the recapitulation section [7]. The passage immediately before the recapitulation, the retransition, provides a window into the nature of musical anticipation. Carl Czerny, a pianist, composer, and music educator in the nineteenth century, gave the following advice to composers: ‘[s]omething [the retransition] of peculiar beauty, calculated to fix the attention, may be introduced in the concluding bars leading back to the principal theme’ [8]. The tension-resolution patterns around the theme’s recurrence in sonata form were found to elicit distinct emotional physiological responses in listeners [9]. In the current study, half of the main themes were of a joyful character and positive valence, and half of a tragic character and negative valence. The addition of tragic stimuli served to reveal behavioral and cognitive mechanisms underlying anticipatory processing of music irrespective of its valence.

The thematic and harmonic resolution at the recapitulation’s start can be regarded as a goal of the listening experience of sonata form. Smith [10] drew parallels between the first-movement sonata form and the Hero’s Journey [11]; the exposition section corresponds to the hero’s departure from home, the development section corresponds to the hero’s expeditions, and the recapitulation section corresponds to the journey’s return stage. The development section ‘is the scene of conflicting moods, of restlessness, of drama, of the unexpected’ [12]. Metaphorically, the main theme finds its way home in the retransition after wandering through remote keys in the development. The German name of the retransition, Rückleitung, means ‘return path’. This path typically emphasizes the dominant of the home key (tonic key). During exposure to a retransition, listeners who are empathetic toward the main theme may recall a goal intention and expect the dominant-tonic resolution, which is the most common type of tension-resolution pattern in music. Therefore, the brain regions implicated in goal-driven attention may be engaged during the concluding bars of retransitions. Based on the literature mentioned above, we hypothesized that the antecedent cues of the main themes in the concluding bars of retransitions were associated with enhanced activity in the premotor and attention control areas.

## 2. Materials and Methods

### 2.1. Participants

The participants were recruited via a public announcement on the internet, which stated that the research goal was to examine the neural responses to sonata form and that a requirement of participants was a high familiarity with Western classical music. To evaluate this requirement, we asked volunteers to express and explain their feelings in response to the musical stimuli in the present study. An online questionnaire was used to collect these data. This questionnaire began with a brief introduction to the musical structure of the first-movement sonata form. The introduction mentioned that (1) this musical form consists of three main sections (i.e., an exposition, a development, and a recapitulation); (2) the themes presented in the exposition recur in the recapitulation; and (3) the passage prior to the recapitulation often intensifies the moving power of the main theme’s recurrence. Eight musical stimuli were presented in a fixed order, with the minor-mode excerpts alternating with the major-mode excerpts. After listening to each stimulus, the volunteers were asked to write down their feelings in response to the retransition and their feelings in response to the main theme’s recurrence. They were also asked to explain their feelings in terms of musical features, such as tempo, tonality/mode, melody, rhythm, timbre, articulation, and dynamics. The volunteers were allowed to listen to the stimulus repeatedly before answering these questions. This online questionnaire was expected to familiarize the volunteers with the experimental stimuli, and the data collected were used for participant screening. The correspondence author of this article selected participants according to the following inclusion criteria: (1) more than five passages immediately before the recapitulation evoked a keen sense of anticipation; (2) the recurrence of more than five themes evoked a feeling of resolution; and (3) their feelings were appropriately explained in terms of musical features for more than five excerpts.

Thirty-one adult volunteers completed this questionnaire. Twenty-seven volunteers met our screening criteria. Twenty participants completed the fMRI experiment. They were free from neurological, psychiatric, or auditory problems and there were no abnormalities in their structural MRI. Fifteen of them had studied musical instruments for six years or more. Written informed consent was obtained from each participant prior to participation in the study. The participants were compensated with approximately USD 16 after the completion of the fMRI scan. Data from two participants were discarded because of excessive head movements. Therefore, the final sample analyzed in this study consisted of 18 participants (12 females aged 27.0 ± 6.7 years, range 20–40 years). All the research procedures were performed in accordance with a protocol approved by the Institutional Review Board of National Taiwan University (201611HM008). This study was conducted in accordance with the Code of Ethics of the World Medical Association (Declaration of Helsinki).

### 2.2. Stimuli

The musical stimuli consisted of four minor-mode and four major-mode excerpts from masterpieces in Western classical music. It was presumed that the minor-mode and major-mode excerpts possess tragic and joyful characters, respectively. The correspondence author of this article (a musicologist) selected the music excerpts from the first movements of famous symphonies and concertos composed in the classical or early romantic period. The following selection criteria for the musical stimuli were used: (1) the first movement was written in sonata form; (2) the tempo was faster than 90 beats per minute; (3) the final passage of the development produced high musical tension; (4) the main theme (first theme) was played at a moderate to high intensity level; and (5) the main theme was of a joyful character (in a major-mode key) or a tragic character (in a minor-mode key). The selected musical stimuli included eight excerpts (detailed in Table S1) around the theme’s recurrence with a duration of 30 s.

It should be noted that the tragic musical themes presented in this study differ from sad music in acoustic, musical, and affective characteristics. Sad music and vocal expressions of sadness such as lamenting, sighing, and weeping are typically soft and slow. On the other hand, tragic themes such as Beethoven’s ‘fate knocking at the door’ theme in his fifth symphony are louder, heavier, and more threatening than sad music. Compared to sad themes, tragic themes are much more frequently used in the first-movement sonata form, which is typically in a moderate to fast tempo. In the present study, the arousing character of the joyful and tragic themes indicates that the arrival of such a theme is a salient musical event.

**Figure 1:**
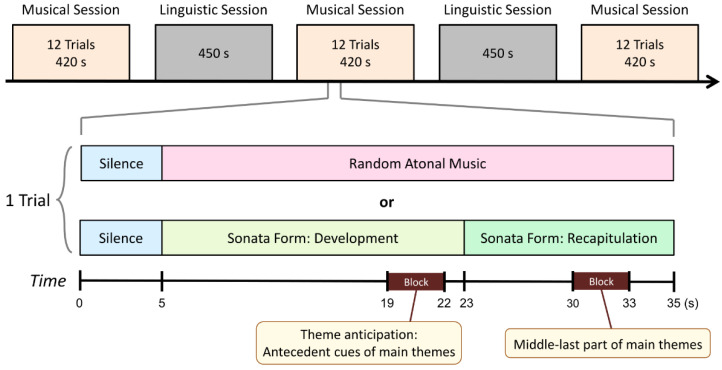
Schematic description of the functional scanning sessions.

All the musical excerpts were extracted from commercially available recordings, digitized (44,100 Hz sampling rate, 16-bit stereo) and edited by GoldWave (version 5.58, Goldwave Inc., Newfoundland, Canada). They were taken from the first movements of symphonies and concertos composed between 1772 and 1858 and written in sonata form. Each excerpt in the present study consisted of an 18 s passage of the concluding portion of the development leading back to the first theme (main theme) and a 12 s passage of the ensuing recapitulation (Figure 1). A brief introduction to these pieces can be found at https://youtu.be/Mz-X7aPcfG8 (accessed on 10 February 2022).

We followed a prior study [13] to use random atonal sequences as stimuli for the control condition. Four atonal random sequences with duration of 30 s were generated using Reason (version 8.0, Propellerhead Software Inc., Stockholm, Sweden). The virtual musical instruments used in these atonal random sequences were the violin, cello, flute, and piano. The duration of each note in these atonal random sequences ranged from 0.1 to 6.0 s, and the fundamental frequency of each note ranged from 65.41 Hz (C2) to 1046.50 Hz (C6). All of the stimuli had a 1 s fade-in and a 1 s fade-out.

### 2.3. Procedure

Prior to the MRI scan, the participants listened to all of the musical stimuli in the same order as that in the online questionnaire. They were asked to rate their agreement with four statements on a seven-point Likert scale (1 = entirely disagree, 2 = mostly disagree, 3 = somewhat disagree, 4 = neither agree nor disagree, 5 = somewhat agree, 6 = mostly agree, 7 = entirely agree) after the presentation of each stimulus: (1) I am very familiar with this music; (2) I felt strong anticipation before the theme came out; (3) the theme is very tragic sounding; and (4) the theme is very joyful sounding.

There were five fMRI scanning sessions separated by 1 min rest intervals, as schematically illustrated in Figure 1. Three fMRI scanning sessions for the present musical study alternated with two fMRI scanning sessions for a linguistic study. This design was expected to minimize affective habituation that could occur with repeated exposure to the same emotional music. The methods and results of this linguistic study [14] are not mentioned further in this paper. Four major-mode music excerpts, four minor-mode music excerpts, and four atonal random stimuli (control) were presented in a pseudo-randomized order within each fMRI scanning session for the present study on musical emotion. The duration of each scanning session was approximately 7 min. Prior to the first scanning session, the first 5 s of Mozart’s Clarinet Concerto were presented to allow the experimenter to adjust the auditory volume according to the participant’s feedback. During this pre-scan volume-adjusting test, the participant also adapted to listening to the music in the presence of scanner noise. The stimuli were presented through scanner-compatible headphones. Participants wore earplugs to reduce the scanner noise by 15–25 dB.

### 2.4. MRI Data Acquisition

The experiments were performed using a Siemens 3T MRI Prisma at National Taiwan University. A gradient-echo echo-planar imaging (EPI) sequence was used in the functional data scanning. Approximately 2.5 mm slices of axial images were acquired in the functional scans using gradient-echo planar imaging with the following parameters: time to repetition = 2500 ms, echo time = 30 ms, flip angle = 87°, in-plane field of view = 192 × 192 mm, and acquisition matrix = 78 × 78 × 45, to cover all the cerebral areas. Magnetization-prepared rapid gradient echo T1-weighted imaging with an isotropic spatial resolution of 0.9 mm was acquired for each participant for spatial individual-to-template normalization.

### 2.5. Data Analyses

We used MIRtoolbox [15] to estimate the sound intensity and brightness of the antecedent cues of the main themes and the middle-last part of these themes in the sonata-form stimuli. Paired *t*-tests were used to examine whether the sound intensity and brightness varied significantly across the antecedent cues of the main themes and the middle-last part of the main themes.

Wilcoxon rank sum tests were used to examine whether the participants’ scores regarding familiarity with musical excerpts, anticipation of all of the themes, the tragic character of the minor-mode themes, the joyful character of the minor-mode themes, the tragic character of the major-mode themes, and the joyful character of the major-mode themes differed significantly from a neutral score of 4. A Bonferroni-corrected significance threshold of *p* < 0.05/6 = 0.0083 was used to account for these comparisons.

The preprocessing and analyses of the fMRI data were performed using SPM12 (Wellcome Trust Centre for Neuroimaging, London, UK) and the Artifact Detection Tools (ART) toolkit. The first four volumes of each run were discarded to allow for magnetic saturation effects. The remaining functional images were corrected for head movement artefacts and timing differences in the slice acquisitions. The preprocessed functional images were coregistered to the individual’s anatomical image, normalized to the standard MNI brain template, and resampled to a 2 mm isotropic voxel size. The normalized images were spatially smoothed using a Gaussian kernel of 5 mm full-width at half maximum to accommodate any anatomical variability across participants. The ART toolkit was used to check the spikes and motion of each dataset. Eighteen functional datasets showed head motion below 2.5 mm of the maximal translation (in any direction), below 1° of maximal rotation throughout the course of scanning, and no outliers occurred. These images were used in the following analyses.

Statistical inference was based on a random effect approach at two levels. The data of each participant were analyzed using the general linear model via fitting the time series data with the canonical hemodynamic response function (HRF) modelled at the relevant blocks in the stimulation paradigm [16]. The block of the antecedent cues of the main themes (theme anticipation) and that of the middle-last part of the main themes are shown in Figure 1. Linear contrasts were computed to characterize responses of interest, averaging across fMRI runs. The group-level analysis consisted of paired *t*-tests for (1) the contrast of theme anticipation minus control, (2) the contrast of the middle-last part of the main themes minus control, and (3) the contrast of theme anticipation minus the middle-last part of the main themes. Results were reported as significant at a voxel-wise threshold level of *p* < 0.001 uncorrected and cluster-level threshold of *p* < 0.05 false discovery rate corrected.

## 3. Results

**Figure 2:**
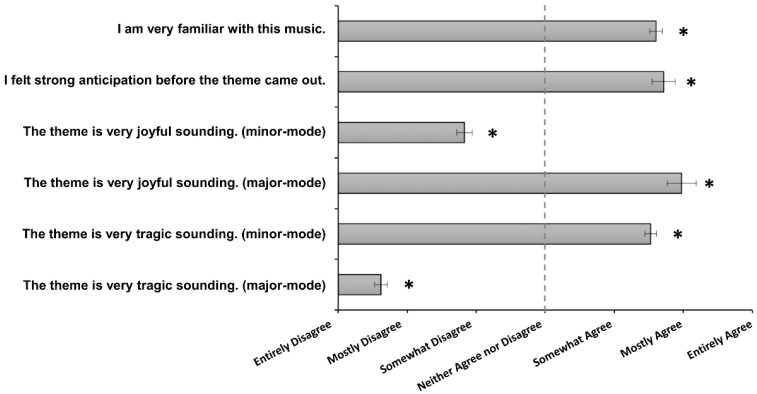
Participants’ ratings (7-point Likert scale). Error bars indicate standard error of the mean. * *p*  <  0.05/6 = 0.0083.

The sound intensity and brightness did not significantly differ across the antecedent cues of the main themes and the middle-last part of the main themes (*p* > 0.5, detailed in Table S1). The results of the questionnaire measures are presented in Figure 2. The Wilcoxon rank-sum tests demonstrated that the scores of familiarity with the musical stimuli, anticipation of the musical themes, the joyful character of the major-mode themes, and the tragic character of the minor-mode themes were significantly higher than a neutral score of 4. By contrast, the scores regarding the tragic character of the major-mode themes and the joyful character of the minor-mode themes were significantly lower than 4. These results showed that the participants were familiar with the musical stimuli and felt strong anticipation before the theme came out. We also confirmed that the minor- and major-mode themes were of tragic and joyful characters, respectively.

**Figure 3:**
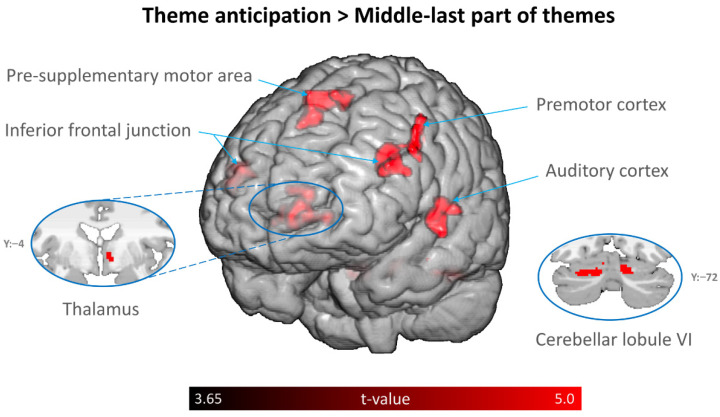
Group-level activation map for the contrast of theme anticipation minus middle-last part of themes.

**Figure 4:**
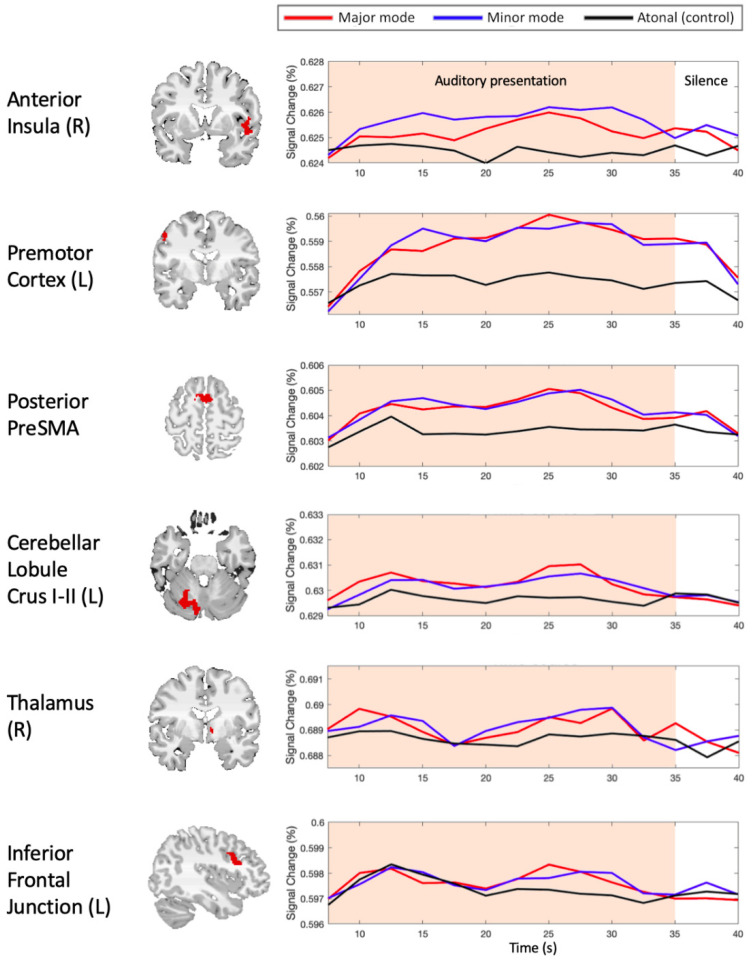
Time courses of focal brain activity in a subset of activated locations. **Left** panels: activation clusters. **Right** panels: averaged time courses of blood-oxygen-level dependent (BOLD) signal change.

The analysis of the fMRI data revealed that the antecedent cues of the main themes (theme anticipation) were associated with significantly greater activity in the bilateral superior temporal gyri, right anterior insula, left putamen, bilateral pre-supplementary motor area (preSMA), bilateral middle frontal gyrus (dorsal premotor cortex), and left posterior cerebellum as compared to the control. The middle-last part of the main themes was associated with significantly greater activity in the anterior portion of the bilateral superior temporal gyri as compared to the control. The right anterior thalamus, right putamen, right caudate, bilateral preSMA, bilateral posterior cerebellum, bilateral inferior frontal junction (IFJ), and left superior temporal gyrus (auditory cortex) showed significantly increased activity for the theme anticipation condition, relative to the middle-last part of the main themes (Table 1, Figure 3). The time courses of focal brain activity in a subset of activated locations, including the anterior insula, premotor cortex, posterior preSMA, posterior cerebellum, anterior thalamus, and IFJ, are demonstrated in Figure 4.

## 4. Discussion

During exposure to familiar music, the listener often attentively tracks the main theme’s recurrences that have structural and affective significance in musical pieces. Motor simulation and attention control processes may be engaged in the cued anticipation of the main theme’s recurrences. To better understand the behavioral and cognitive mechanisms underlying this anticipation, we used fMRI to examine the neural responses to the concluding bars of retransitions in sonata form. A retransition encompasses musical cues that foster the listener’s motivation and cognitive organization of the main theme’s recurrence. Compared to the control condition of atonal random music, the cued anticipation of the main themes during the retransitions was associated with enhanced activity in the anterior insula, premotor regions, and posterior cerebellum. This finding bore a remarkable similarity to the network that commonly subserves predictive coding in action perception, language, and music [17]. Moreover, we observed significantly enhanced activity in the anterior thalamus, putamen, caudate, preSMA, premotor cortex, posterior cerebellum, IFJ, and auditory cortex during theme anticipation compared to the middle-last part of the main themes.

A novelty of the present study was the use of both positively and negatively valenced musical excerpts as stimuli. The greater activity in the right anterior thalamus during the anticipation of joyful and tragic main themes than during the middle-last part of these themes may reflect its role in the anticipation of salient events. The right anterior thalamus has been found to be involved in itch processing [18], anticipation of monetary reward [19,20,21], anticipation of aversive pictures [22], and anticipation of pain [23]. Moreover, animal experiments demonstrated that memory tasks for temporally linked items are sensitive to anterior thalamus damage. For example, lesions of the anterior thalamus in rats impaired their ability to remember the sequential order of a series of odors in order to retrieve food rewards [24]. There is emerging evidence indicating that the anterior thalamus may guide attention towards stimuli that consistently predict important outcomes [25]. Our finding of the involvement of the right anterior thalamus in the cued anticipation of salient musical themes lends support to the role of the anterior thalamus in anticipatory processing of motivationally salient events. It should be noted that emotional responses to the anticipation of joyful and tragic themes could be elusive and divergent, and therefore, we did not observe significant activation in the amygdala or nucleus accumbens for theme anticipation.

Since the participants were familiar with the sonata-form stimuli, they were likely to track the sequence of antecedent cues and theme recurrence via covert humming along with music. As expected, we observed greater activity in the premotor cortex, preSMA, and posterior cerebellum during the theme anticipation condition relative to the control stimuli. These areas have been implicated in covert or overt rehearsal of familiar music [26,27,28] and melody imagery [29]. 

The preSMA and posterior cerebellum showed greater activity for the theme anticipation condition than for the middle-last part of the main themes. It is well recognized that the preSMA supports domain-general sequence processing [30]. The posterior part of the preSMA underpins linear sequence processing in language [31] and music [32]. In the present study, each cue–theme sequence was fixed and linear. Therefore, the increased activity in the posterior (y~2) preSMA for theme anticipation may reflect its role in linear sequence processing. On the other hand, the anterior (y~13) preSMA has been implicated in working memory processes involved in sentence comprehension [33] and motor response selection/control [34]. In the present study, the participants may have recruited the anterior preSMA for maintenance of the ensuing theme during the final bars of the retransition, while the working memory load may have significantly decreased after the entry of this theme. Moreover, the greater activity in the anterior preSMA during theme anticipation may have reflected more vivid imagery of the music in service of attentively monitoring the theme’s recurrence, compared to the middle-last part of the main themes. It is proposed that the preSMA may support the generation of rich and fine-grained sensory expectations when the task requires attentive access to auditory representations [35]. Increased activity in the anterior preSMA has been linked to the attentive monitoring of goal progress over time [36]. Taken together, these observations allow us to suggest that the preSMA underpins the linear sequence processing and vivid fine-grained musical imagery necessary for precisely monitoring the theme’s recurrence.

The cerebellum is implicated in the prediction of sensory outcomes as a consequence of motor command, and this predictive computation is known as an internal forward model [37]. During motor learning or motor control, the cerebellum is engaged in the fine modulation of the movement parameters controlled by the cortex [38]. Moreover, the posterior cerebellum provides prospective signals about the timing of perceptual events [39]. Increased activity in the posterior cerebellum during theme anticipation suggests that this region may utilize such prospective signals to make fine adjustments to covert humming along with the music. This view is consistent with a study on joint actions showing that the posterior cerebellum processed errors in synchrony between one’s own actions and those of others [40]. Like joint actions, humming along with familiar music may recruit the posterior cerebellum to fine tune the way in which the listener needs to coordinate the imagined action with the auditory inputs.

An interesting finding of the present study was the involvement of the bilateral IFJ in theme anticipation. Accumulating evidence suggests that the IFJ plays an essential role in tasks requiring cognitive control such as working memory and task switching [41,42,43]. Two studies suggest specific aspects of the hub role of the left IFJ. De Baene and colleagues [44] indicated that the left IFJ may contribute to the integration of goal information and action-related information. Asplund and colleagues [45] pointed out that the left IFJ may orchestrate goal-directed attention control and stimulus-driven attention. In the present study, the goal-driven attention linked to the harmonic/thematic resolution at the recapitulation’s start and the attention allocated to covert humming along with music might meet in the IFJ. While this hypothesis must presently be viewed as speculative, it provides a novel view on the role of the inferior frontal regions in the anticipatory processing of music. In a putative network of predictive coding, the bilateral inferior frontal gyri were reported to underpin the processing of harmonic syntax, the recognition of structural regularities, the analysis of structural relationships among events occurring within sequences, and the hierarchical organization of actions during multi-component goal-directed behavior [17]. We suggest that the coincidence of the release of harmonic tension and the main theme’s recurrence at the recapitulation’s start may be a goal of the listening experience of sonata form. The pursuit of this goal may engage a suite of cognitive and audiomotor processes, including goal-driven attention allocation, maintenance of the goal in working memory, and monitoring the progress toward the goal via re-enactment of the fixed sequence of the cues and the main theme. The bilateral IFJ might be a hub for driving and coordinating these processes.

When comparing the theme anticipation condition to the middle-last part of the main themes, exclusively left-sided activation was observed in the auditory cortex and dorsal premotor cortex. It has been found that responses of the human auditory cortex to rapid temporal information were weighted towards the left [46,47]. Moreover, relative to the connections of the right dorsal premotor cortex, those of the left dorsal premotor cortex were stronger with frontal and parietal areas representing action dominance based on a lead in motor intention and fine precision skills [48]. In light of these findings, the sensitivity of the left auditory cortex and left dorsal premotor cortex to the goal-directed anticipation of music might be related to their role in providing goal-relevant, fine-grained audiomotor representations on a narrower time scale. It is noteworthy that our finding of increased activity in the left auditory cortex and attention control regions for theme anticipation compared to the middle-last part of the main themes supports the idea that attention modulates early sensory processing, somewhat speaking against the idea that expectation has little influence on early sensory responses [49]. While there have been a number of studies on visual selective attention and visual expectation, the present study calls for future studies to illustrate how goal-directed anticipation, attention, and motor resonance modulate activity in the auditory cortex in response to familiar musical passages.

**Figure 5:**
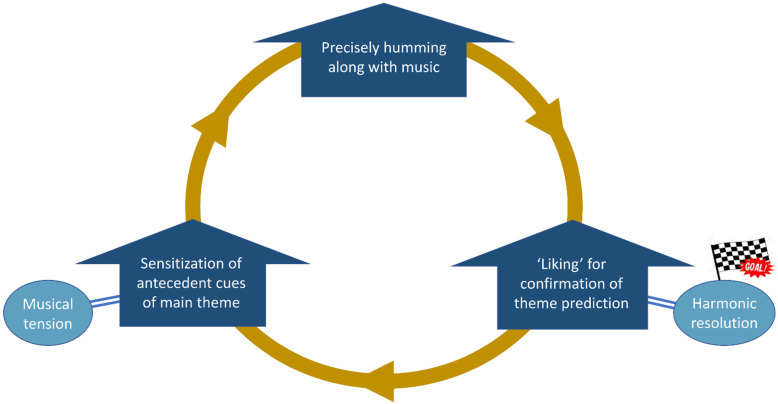
Putative model for the enjoyment and learning of music (inspired by the incentive-sensitization theory of addiction). During exposure to a musical piece, the antecedent cues of the main theme may increase attention to covert humming along with the music. More precise humming may lead to more intense ‘liking’ for the confirmation of theme prediction. Repeated intermittent experiences of ‘liking’ may in turn cause sensitization to the antecedent cues of the main theme. The listener’s ‘wanting’ and ‘liking’ may be facilitated by the musical tension of the antecedent cues and the harmonic resolution at the main theme’s entry, respectively.

In light of the incentive-sensitization theory of addiction [50], our results might provide important information for the mechanisms underlying the enjoyment and learning of music. A core of enjoyment of familiar music might be incentive sensitization to cues, namely, amplification of ‘wanting’ for the main theme triggered by its antecedent cues. Attentively monitoring the sequence of the antecedent cues and the main theme might be rewarded with pleasure in the brain when this prediction is confirmed by the auditory inputs. Importantly, the confirmation of this prediction might be more highly rewarded when this prediction is based on more precise, fine-grained audiomotor representations. During the process of familiarization with a specific musical piece, the listener might undergo a cycle where sensitization to the antecedent cues of the main theme, attentively and precisely humming along with the music, and ‘liking’ for the confirmation of the prediction of this theme enhance each other. The musical tension of the antecedent cues and the harmonic resolution at the main theme’s entry could contribute to the listener’s ‘wanting’ and ‘liking’ in this cycle, respectively (Figure 5). Given that the anteromedial thalamus may play a role in instrumental learning processes and the control of goal-directed behavior [25], our finding regarding the involvement of this region in theme anticipation provides preliminary support for this hypothesis. Future studies could consider using a paradigm of learning cue–theme sequences to test this hypothesis.

There were several limitations of this study that need to be pointed out. First, the final sample of the fMRI data consisted of 18 participants, and the sonata-form stimuli were extracted from only eight compositions. Future research should increase the sample size of participants and musical stimuli. Second, the present study lacked behavioral data about the participants’ affective/motivational states and the vividness of the auditory imagery during fMRI scanning. We did not ask the participants to respond after stimulus presentation because we were concerned about the distracting effect of self-monitoring for rating tasks. Third, we did not analyze the IFJ’s functional connectivity to the thalamus, premotor regions, posterior cerebellum, or auditory cortex. Therefore, the IFJ’s specific contributions to driving and coordinating cognitive and audiomotor processes remain unclear. We suggest that future research could be devoted to the parcellation of the bilateral IFJ using musical stimuli imbued with incentive salience.

In conclusion, the present study focused on listeners’ cued anticipation of the main theme’s recurrence. While partially consistent with the putative prediction network underlying action perception, language, and music [17], our results demonstrate parallels between attentively monitoring the main theme’s recurrence and goal pursuit. Given that the harmonic/thematic resolution at the recapitulation’s start serves as a goal of the listening experience of sonata form, the anterior thalamus may play a key role in guiding attention towards the antecedent cues of this goal. The pursuit of the main theme’s recurrence was associated with increased activity in the posterior cerebellum and premotor areas, which may contribute to monitoring the progress toward a goal via fine-grained motor simulation and maintaining the goal in working memory. The bilateral IFJ might orchestrate the attention allocation to motor simulation and goal-driven attention. These findings highlight how cognitive and audiomotor processes work together during the cued anticipation of motivationally salient musical events.

## Figures and Tables

**Table 1:** Activation clusters for the contrasts of theme anticipation minus control, middle-last part of themes minus control, and theme anticipation minus middle-last part of themes.

	*t*-Value	MNI Coordinates (mm)			Cluster Size (Voxel)	Brain Regions
		X	Y	Z		
Theme Anticipation > Control	8.55	−48	0	−2	190	Superior temporal gyrus
	5.90	−48	−34	12	73	
	6.87	50	6	−8	260	Superior temporal gyrus
	6.81	48	0	0		Insula
	4.33	56	10	−14		Superior temporal pole
	8.00	−24	0	6	79	Putamen
	7.45	50	−2	56	251	Middle frontal gyrus (premotor cortex)
	7.28	8	10	62	241	Pre-supplementary motor area
	5.62	−32	−56	−30	143	Cerebellar lobule VI
	5.32	−52	0	42	125	Middle frontal gyrus (premotor cortex)
Middle-last Part of Themes > Control	6.8	−50	0	−8	145	Superior temporal gyrus
	5.93	52	−2	−10	96	
Theme Anticipation > Middle-last Part of Themes	6.81	−6	2	64	364	Pre-supplementary motor area
	6.62	−46	−16	0	175	Superior temporal gyrus
	5.74	14	−72	−24	151	Cerebellar lobule VI
	5.32	12	−80	−26		Cerebellar lobule Crus I-II
	5.63	−10	−80	−28	240	Cerebellar lobule Crus I-II
	5.32	−14	−72	−26		Cerebellar lobule VI
	5.66	46	20	26	119	Inferior frontal gyrus, pars opercularis (inferior frontal junction)
	5.63	46	18	22		Inferior frontal gyrus, pars triangularis (inferior frontal junction)
	5.65	−36	14	32	289	Inferior frontal gyrus, pars opercularis (inferior frontal junction)
	5.32	−42	14	28		Inferior frontal gyrus, pars triangularis (inferior frontal junction)
	4.80	−52	8	44		Middle frontal gyrus (premotor cortex)
	5.62	8	−2	0	342	Anterior thalamus
	5.42	22	10	2		Putamen
	5.39	22	10	8		Caudate
	5.05	20	−62	−24	93	Cerebellar lobule VI

Abbreviations—MNI: Montreal Neurological Institute coordinate system.

## Data Availability

The data are not publicly available due to privacy/ethical restrictions. The data presented in this study are available on request from the corresponding author.

## Supplementary Materials

The following supporting information can be downloaded at: https://www.mdpi.com/article/10.3390/brainsci12020261/s1, Table S1: Sonata-form stimuli.
