# Supplementary material for: Attention Control and Audiomotor Processes Underlying Anticipation of Musical Themes while Listening to Familiar Sonata-Form Pieces
Source: Brain Sci. 2022 Feb 13;12(2):261. doi: 10.3390/brainsci12020261 (PMC8870438; doi:10.3390/brainsci12020261)
Supplement: Supplementary file 1 [file brainsci-12-00261-s001.zip › brainsci-1587133-supplementary.pdf]

Supplementary Material

Attention control and audiomotor processes underlying anticipation of musical themes while listening to familiar sonata-form pieces

Table S1. Sonata-form stimuli.

Key mode	Composer	Work	Performers	Intensity (root mean square)		Brightness	
				Antecedent cues of theme	Middle-final portion of theme	Antecedent cues of theme	Middle-final portion of theme
Major mode	Wolfgang Amadeus Mozart	Concerto for Flute, Harp, and Orchestra in C major, K. 299/297c	Academy of St. Martin-in-the-Fields conducted by Sir Neville Marriner. Soloists: Irena Grafenauer (flute) and Maria Graf (harp).	0.203	0.067	0.261	0.197

	Felix Mendelssohn-Bartholdy	Symphony No. 4 (‘*Italian*’) in A major, Op. 90	London Symphony Orchestra conducted by Claudio Abbado.	0.192	0.097	0.352	0.441
	Ludwig van Beethoven	Symphony No. 7 in A major, Op. 92	Cleveland Orchestra conducted by George Szell.	0.116	0.150	0.369	0.304
	Ludwig van Beethoven	Piano Concerto No. 5 (‘*Emperor*’) in E-flat major, Op. 73	Berliner Philharmoniker conducted by Ferdinand Leitner. Soloist: Wilhelm Kempff (piano).	0.197	0.231	0.361	0.295
Minor mode	Franz Joseph Haydn	Symphony No. 45 (‘*Farewell*’) in F-sharp minor, Hob. I:45	Orchestra of St. Luke’s conducted by Sir Charles Mackerras.	0.019	0.103	0.483	0.357
	Wolfgang Amadeus Mozart	Symphony No. 25 in G minor, K. 183/173dB	Wiener Philharmoniker conducted by Leonard Bernstein.	0.070	0.159	0.214	0.521
	Ludwig van Beethoven	Symphony No. 5 (‘*Fate*’) in C minor, Op. 67	Wiener Philharmoniker conducted by Carlos Kleiber.	0.120	0.027	0.425	0.260

	Johannes Brahms	Piano Concerto No. 1 in D minor, Op. 15	Berliner Philharmoniker conducted by Sir Simon Rattle. Soloist: Krystian Zimerman (piano).	0.166	0.088	0.362	0.189
